# Diseases of the Ear, Nose, and Throat, and Their Accessory Cavities

**Published:** 1898-03

**Authors:** 


**Diseases of the Ear, Nose, and Throat, and their Accessory Cavities.** By SETH SCOTT BISHOP, M.D. Pp. xv., 496. Philadelphia: The F. A. Davis Company. 1897.

It is not uncommon at the present time to meet with text-books combining the subjects of ear, nose, and throat diseases, and we have nothing to say against this plan, inasmuch as they are very closely associated; while in clinical departments of hospitals, where students acquire their knowledge of these subjects, they are very frequently combined. The present work contains in a moderate compass a good condensed account of these affections, so as to constitute it a key or introduction to the exhaustive treatises which we possess on each topic separately. Several subjects are treated in greater detail than characterises the work as a whole: these being diphtheria, with its antitoxin treatment; surgical operations about the mastoid process; and the treatment of middle-ear affections by compressed air. These are all modern developments, and cannot be found fully treated in older works, hence we think the author has done wisely in giving them ample space. The chapters on diphtheria are excellent. A good account is given of the Klebs-Löffler bacillus, and the best means of diagnosing it from other similar micro-organisms; while a full account of the treatment by antitoxin, with its risks as well as its successes, is added. The author gives us one of the best accounts of the operations about the mastoid process that we have seen in any text-book. His descriptions are very clear, and are illustrated by photographs taken directly from life or from the cadaver so as to ensure absolute accuracy. We are not favourably impressed by the author's method of treating middle-ear affections by compressed air. He is not content with the ordinary Politzer's bag, nor with the common method of inflation through the Eustachian catheter; but recommends instead the use of a reservoir armed with a pressure gauge, from which compressed air is allowed to issue through a stop-cock and pass through a close-fitting nose-piece into the nasal cavity, with the patient's nose firmly closed and cheeks fully distended. He recommends a pressure varying from a few pounds in infants up to sixty or eighty pounds in old, thickened, and hardened drum-heads. We should be very chary ourselves of using a method such as this, which must admit the possibility of considerable damage being occasionally done; and we altogether fail to see the advantage it possesses over older and safer methods. The book is one which can be safely recommended as a whole, and will amply repay a careful perusal.

